# Supplementary material for: MetaRibo-Seq measures translation in microbiomes
Source: Nat Commun. 2020 Jun 29;11:3268. doi: 10.1038/s41467-020-17081-z (PMC7324362; doi:10.1038/s41467-020-17081-z)
Supplement: Supplementary file 10 — Supplementary Data 7 [file 41467_2020_17081_MOESM10_ESM.zip › File2/Confidence_High_Taxonomy/144007_out.krona.html]

Javascript must be enabled to view this page.

members
magnitude
magnitudeUnassigned
count
unassigned
taxon
rank

144007\_out

3

3
superkingdom
2

phylum
1239
3

186801
class
3

3

SRS057478\_contig\_number\_8073
186802
1
order

172733

SRS015431\_contig\_number\_contig-100\_42408.129562
species
1

1
216572
family

459786
genus
1

1

SRS149181\_contig\_number\_12365
876091
species
